# Development and validation of a risk prediction model for lost to follow-up among adults on active antiretroviral therapy in Ethiopia: a retrospective follow-up study

**DOI:** 10.1186/s12879-022-07691-x

**Published:** 2022-09-07

**Authors:** Dawit Tefera Fentie, Getahun Molla Kassa, Sofonyas Abebaw Tiruneh, Achenef Asmamaw Muche

**Affiliations:** 1 Department of Epidemiology and Biostatistics, Institute of Public Health, University of Gondar, Gondar, Ethiopia; 2 Department of Public Health, College of Health Sciences, Debre Tabor University, Debre Tabor, Ethiopia

**Keywords:** Lost to follow up, HIV/AIDS, Risk prediction, Risk score, Regression formula

## Abstract

**Background:**

Over 420,000 people have initiated life-saving antiretroviral therapy (ART) in Ethiopia; however, lost-to-follow-up (LTFU) rates continues to be high. A clinical decision tool is needed to identify patients at higher risk for LTFU to provide individualized risk prediction to intervention. Therefore, this study aimed to develop and validate a statistical risk prediction tool that predicts the probability of LTFU among adult clients on ART.

**Methods:**

A retrospective follow-up study was conducted among 432 clients on ART in Gondar Town, northwest, Ethiopia. Prognostic determinates included in the analysis were determined by multivariable logistic regression. The area under the receiver operating characteristic (AUROC) and calibration plot were used to assess the model discriminative ability and predictive accuracy, respectively. Individual risk prediction for LTFU was determined using both regression formula and score chart rule. Youden index value was used to determine the cut-point for risk classification. The clinical utility of the model was evaluated using decision curve analysis (DCA).

**Results:**

The incidence of LTFU was 11.19 (95% CI 8.95–13.99) per 100-persons years of observation. Potential prognostic determinants for LTFU were rural residence, not using prophylaxis (either cotrimoxazole or Isoniazid or both), patient on appointment spacing model (ASM), poor drug adherence level, normal Body mass index (BMI), and high viral load (viral copies > 1000 copies/ml). The AUROC was 85.9% (95% CI 82.0–89.6) for the prediction model and the risk score was 81.0% (95% CI 76.7–85.3) which was a good discrimination probability. The maximum sensitivity and specificity of the probability of LTFU using the prediction model were 72.07% and 83.49%, respectively. The calibration plot of the model was good (p-value = 0.350). The DCA indicated that the model provides a higher net benefit following patients based on the risk prediction tool.

**Conclusion:**

The incidence of LTFU among clients on ART in Gondar town was high (> 3%). The risk prediction model presents an accurate and easily applicable prognostic prediction tool for clients on ART. A prospective follow-up study and external validation of the model is warranted before using the model.

**Supplementary Information:**

The online version contains supplementary material available at 10.1186/s12879-022-07691-x.

## Introduction

Human immunodeficiency virus (HIV) is a significant global public health issue claiming almost 33 million lives so far [1]. With increasing access to effective HIV programs, HIV infection has become one of manageable chronic health condition, enabling people living with HIV to lead long and healthy lives [2]. However, ART programs in low and middle-income countries (LMIC) are characterized by high rates of loss to follow-up from care (LTFU), up to 40% after 5 years of ART [3]. Thus, LTFU is threatening optimal standard achievement in attaining near-universal ART coverage.

The United Nations acquired immunodeficiency syndrome (UNAIDS) fast-track goals, commonly referred to as the 95-95-95 goals, recommend countries should have 95% of HIV-infected persons know their HIV status, 95% of those who know their HIV positive status should initiate ART and 95% of those on treatment should have viral suppression (< 1000 copies/ml) by 2030 to control the global HIV pandemic [4].

Good adherence to ART treatment is essential in achieving viral suppression and reduction in HIV transmission [5]. However, many countries in sub-Saharan Africa (SSA) still suffer from high rates of LTFU on ART, poor adherence to treatment, and low retention rates. A systematic and meta-analysis research which was done in LMIC with a total sample size of 1,605,320 clients of which 87.4% where from SSA has reported the percentage of patients identified as LTFU varied between 2.8 and 65.6% [6]. In Ethiopia, currently there are around 420,000 people on ART [7], while a systematic and meta-analysis study has revealed a large proportion (11.86% to 18.47%) of clients on ART were LTFU [8].

Knowledge of risk factors for LTFU can contribute to improve individualized patient care and inform policymakers on a programmatic level. Let alone the rest part of the world, in recent years, a substantial number of studies in Ethiopia have revealed a wide range of sociodemographic male sex [9–16], rural residence [17–20], older age [18, 21, 22], type of work-being daily laborer [18, 21, 23], educational status [15, 22–24], and unmarried [15, 23], clinical parameters (underweight BMI [9, 22, 25], type of regimen [9, 21, 24], lower CD4 cells [14, 23, 25–27], advanced WHO staging [12, 17, 26–28], poor drug adherence [18, 25, 27], not taking prophylaxis isoniazid [25, 27–29] and/or cotrimoxazole preventive therapy [20, 21, 30], presence of opportunistic infection [25, 26, 29], bed-redden patients [12, 21, 25–27], having adverse drug reaction [28, 29], and mental illness [19, 20], personal and behavior-related (absence of care giver [19, 20], substance abusers [23, 31], not disclosing HIV status [11, 30] and fear of stigma [19, 24] and health system-related (less burden health facilities [14, 16, 22, 26] determinants that are independently associated with LTFU.

Despite the significant number of individual studies on incidence and predictors of LTFU, to this date and to our knowledge, there is no means or tool to identify patients at higher risk for LTFU among clients on ART. Previous studies concluded that risk factors for LTFU in HIV care are known, but individual prediction tools are lacking [32], and predicting lost to follow-up using routinely collected data was not successful [33]. Consequently, developing a prediction tool would help in differentiating such patients at higher risk of LTFU and thus, it would assist health professionals in providing special care for such patients and minimizing LTFU along with preventing the grave consequences following it. We believe that if clinics with limited resources could easily use an accurate tool for predicting LTFU, might help them modify services that would optimize health care delivery. Thus, our goal was to develop and validate a practical clinical prognostic risk prediction tool that will use routinely collected data from HIV clinics to predict LTFU among clients on ART.

## Methods and materials

### Study design, area, and period

An institutional-based retrospective follow-up study with a prognostic approach was conducted from October 2016 to April 2021 among adult clients on ART among health facilities in Gondar town. Gondar town is the main city of the central Gondar zone located 750 km away from Addis Ababa, the capital city of Ethiopia. The town has eight government health centers and one specialized referral hospital (University of Gondar compressive and specialized hospital) which provides acute and chronic HIV/AIDS care. The University of Gondar compressive and specialized hospital is a teaching hospital that serves more than seven million people of the central Gondar zone and people of the neighboring zones.

### Source and study populations

The source population includes all adult (age 18 years and above) HIV-positive patients who have started ART and have a follow-up in Gondar town health facilities from October 2016 (starting the universal test and treat strategy) to April 2021. The study population was those clients on ART from the University of Gondar compressive and specialized hospital, Gondar health center, and Marakie health center from the start of the universal test and treat strategy in the town (October 2016 to April 2021). We have included all adult (age 18 years and above) HIV positive patients on ART in Gondar town from October 2016 to April 2021 and those patients with incomplete information for outcome variable (lost to follow up, dead, drop, transfer out) and ART initiation was excluded from this study.

### Sample size determination and sampling technique

The sample size was determined by using the formula

$${\text{N}} = \left( {{1}.{96}/\delta } \right)^{{2}} \emptyset ({1} - \emptyset )$$

where N is the total sample size, δ is the margin of error (≤ 0.05), and Ø is the outcome proportion [34]. Accordingly, the total sample size was determined to be 334 and 236 using the proportion of 32% [25] and 19% [21], respectively which was taken from two studies done in one of the study setting considered in this study (University of Gondar comprehensive and specialized hospital). Nevertheless, to increase the accuracy of the prediction model, a larger sample size (N = 432) was taken. A total of 432 patients from three different facilities with computer-generated simple random sampling technique with the help of smart care program were considered for prediction of LTFU from the ART chart book at Gondar town.

### Variables of the study

Lost to follow up from ART care was the predicted variable. Age (18–50 years versus greater than 50 years), sex, marital status (married versus unmarried (single, widowed, and divorced), educational status (formal vs non-formal), religion (Christian (Protestant, Orthodox) versus Muslim), and residence (rural and urban) were sociodemographic prognostic determinants. Whereas, partner HIV status (known vs unknown), HIV disclosure status (yes or no), and presence/absence of caregiver were considered as behavioral prognostic determinates. Furthermore, clinical determinants such as time of ART initiation (same day versus not same day), Active TB disease (presence/absence), baseline WHO staging (stage I/II versus advanced stage-III/IV), BMI (underweight versus normal versus obese), Functional status (bedridden and ambulatory versus working), prophylaxis status (on prophylaxis (INH and/or CPT) vs not on prophylaxis), viral load status (HVL versus not HVL), and appointment spacing model (ASM) status (on ASM versus not on ASM) were considered. Referral site (referred from inside facility versus outside facility) was other prognostic determinant.

### Operational definitions

Loss to follow up (LTFU): A patient who has not been seen at the clinic for at least 30 to 90 days (3 months) after the last missed appointment, but has not transferred out or dead.

Defaulter: is a client who has not turned up for either a clinical visit or refill 7 days after their scheduled appointment date but is not a patient classified as LTFU.

Drop a client who has not turned up or come back to the clinic for either a clinical visit or refill for more than 90 days (3 months) from the last scheduled visit.

Time to LTFU: Time to LTFU was calculated in years according to the time interval between the dates of ART initiation to LTFU.

Rapid ART initiation: defined as starting ART within 7 days.

Late ART initiation: defined as starting ART after 7 days of HIV diagnosis.

Transfer Out (TO): Refers to the date on which a patient who has been receiving ART at one facility transfers out of that facility and receives the treatment at other facilities.

Dead (D): A patient who died at any time after being enrolled in HIV care.

Non-adherent: patients who are labeled as having “Poor” or “Fair” adherence in ART follow up card.

Adherent: patients who are labeled as having “Good” adherence in ART follow-up card.

High viral load (HVL): patients with a viral load above 1000 copies/ml after 6 months of initiation of ART.

### Data collection procedure and quality assurance

The method of data collection and data analysis plan was adapted from our previous prediction study “Methods and materials” section [35]. The available information on the patient records and literature had been first observed and an appropriate data extraction tool was prepared in English. The data extraction tool was pre-tested to understand the review tools and completeness of data items on 15 charts at the same facility as it is secondary data and the necessary amendment was made to the final data extraction format. Then the data was collected by four B.Sc nurses who had ART training using the prepared data collection format on the already existing records after half day theoretical and half-day practical training given on the study’s objective and how to retrieve data for the study purpose using the data extraction format. They were also briefed on the definition of variables in the questionnaire and registration charts. One data clerk also supported them by identifying the charts. Charts were retrieved using the patient’s registration number and unique ART number which was found in a database in the electronic system (smart care). The retrieval process was closely monitored by the principal investigator throughout the data collection period. The collected questionnaires were checked regularly for completeness of the information upon arrival, and any gaps identified were immediately communicated to the data collectors for possible correction.

### Data management and analysis

The data were entered using Epi-Data version 4.6 software. The data was analyzed using R-programming version 4.0.3 software. Descriptive statistics including tables, mean with standard deviations (SD) for normally distributed continuous variables, percentages, and rates were employed. Incidence of lost follow was computed. First, binary logistic regression was fitted to see the association of each potential determinant with the incidence of LTFU among patients on ART. All variables with a p-value < 0.25 in the bi-variable analysis were included in the multivariable model. As well, least absolute shrinkage and selection operator (LASSO) regression was considered for variable selection for the multivariable model. The two-sided p-value less than 0.05 was considered statistically significant. So that the final multivariable binary logistic regression model (logit) was considered as the following model equation.

$${\text{Logit }}\left( {\text{y}} \right) = \beta_{0} + \beta_{{\text{i}}} {\text{ X}}_{{\text{i}}} + \cdots \beta_{{\text{k}}} {\text{X}}_{{\text{k}}}$$

where y is the binary dependent variable (Lost follow-up or not), β_0_ is the constant when all predictors are equated to zero, β_i_ is the ith coefficient for determinants i, i = 1, 2, 3…, k. x_i_ is the ith determinant variable.

### Assessment of the model performance and validation

Model calibration was assessed by plotting deciles of the predicted probability of lost follow-up against the observed rate of LTFU in each decile and fitting a smooth line. The AUROC curve was done to see the model discrimination probability using ‘pROC’ and the calibration plot was checked using ‘givitiR’ R-packages. The AUROC value of 0.5 indicates no predictive ability while 0.7 and above is considered as good and one is perfect prediction probability. The regression coefficients with their 95% confidence intervals and AUROC were internally validated using the binormal smoothing bootstrapping technique. The bootstrap method with 2000 iterations of re-samplings with replacements to create bootstrap datasets and bootstrapping was used to adjust for optimism/overfitting in the predictive ability of the model. After bootstrapping, the model’s predictive performance was considered the performance that can be expected when the model is applied to future similar populations. To evaluate the clinical and public health impact of the model, we performed a decision curve analysis (DCA) of standardized net benefit across a range of threshold probabilities (0 to 1). In the DCA, the model was compared against two extreme scenarios; “intervention for all” and “no intervention”. In our case, the intervention considered was using the model for the prediction of LTFU for all patients.

### Prognostic individualized prognostic risk prediction development

To construct an easily applicable score chart rule was used. The predicted probability of LTFU was presented according to two categories of the risk score for statistical stability and practical applicability using the Youden index value. The categories were chosen with a view to a reasonable size of each type and clinical sensibility, and the classification was high or low risk for LTFU. The risk score category was categorized using sensitivity, specificity, the positive and negative predictive value of the risk score model using different cut-point values. Finally, the probability of LTFU for each patient on ART was predicted using the linear predictor of estimated risk of lost follow up which is:

$${\text{P }}\left( {\text{Risk score for each patient}} \right) = {\text{risk score }} \times {\text{ prognostic determinant }} + \cdots + {\text{ N }}\left( {\text{risk score}} \right) \, \times {\text{ N }}\left( {\text{prognostic determinant}} \right)$$

As well, for the ease of clinical preference the individualized risk prediction was classified also using a regression formula based on the Bernoulli distribution formula.

$${\text{Logit}}\left( \pi \right) = \alpha \, \beta {\text{x}} \ldots + + + \, \ldots \, \beta_{{\text{n}}} {\text{X}}_{{\text{n}}} .$$

## Results

### Socio-demographic variables of the study participants

Out of 432 study subjects, 230 (53.24%) were females. The mean age was 33.83 years with a Standard deviation (SD) ± 10.04 years. Most participants were Orthodox Christian (382, 88.43%), and more than half were unmarried (261, 60.42%). The majority of patients were from Gondar town (urban) (353, 81.71%). More than two-thirds of patients (309, 71.53%) had formal education and more than half (228, 53.78%) were employed. Concerning disclosure, 236 (54.63%) patients did not disclose their HIV status (Table 1).

**Table 1:** Baseline socio-demographic characteristics of adult clients on ART in Gondar town, Oct 2016 to April 2021 (n = 432)

Characteristics	Frequency (n)	Percentage (%)
Age (years)		
18–50	380	87.96
> 50	52	12.04
Sex		
Male	202	46.76
Female	230	53.24
Marital status		
Never married	103	23.84
Married	171	39.58
Divorced	131	30.32
Widowed	27	6.25
Level of education		
No formal education	123	28.47
Primary	123	28.47
Secondary	130	30.09
Tertiary	56	12.96
Religion		
Muslim	44	10.19
Orthodox	382	88.43
Protestant	6	1.39
Occupation		
Employed*	228	52.78
Unemployed**	204	47.22
Caregiver		
Family^#^	314	72.69
Non-family^#^*	76	17.59
No caregiver	42	9.72
Residence		
Within Gondar town (urban)	353	81.71
Out of Gondar town (rural)	79	18.29
Disclosure status		
Disclose	196	45.37
Not disclose	236	54.63

^*^Employed—private employee, driver, Governmental employee, Teacher, Merchant, Military

^**^Unemployed—Housewife, Student, Farmer, unemployed, daily worker

^#^Family—patient’s husband or wife, child or brother or sister or parents

^#^*Non-family caregiver outside “family” definition

### Baseline clinical characteristics of patients at ART initiation

One-third of patients knew their HIV status and has started ART in the same facility where they were diagnosed. Along with ART, nearly two-thirds of patients (281, 65.05%) were also put on prophylaxis (CPT 11.11%, INH 37.96%, on both (CPT and INH), 15.97%) while more than one-third of patients (151, 34.95%) were not taking prophylaxis because they were not eligible as per the national guideline which was documented in their respective charts. The majority of patients (317, 73.38%) were adherent to their medication. Since the current strategy is “test and treat”, the majority of patients (366, 84.72%) were working and were not having advanced diseases (WHO stage III/IV) (368, 85.19%) while they were starting ART (Table 2).

**Table 2:** Baseline clinical characteristics of adult HIV-positive patients at initiation of ART in Gondar town, October 2016 to April 2021 (n = 432)

Characteristics	Frequency	Percentage (%)
Time of initiation of ART ASM		
Same day	269	62.3
Not same day	163	37.7
Yes	357	82.6
No	75	17.4
Partner HIV status		
Positive	78	18.1
Negative	49	11.3
Unknown	305	70.6
TB status at ART initiation		
Yes	45	10..4
No	387	89.6
Prophylaxis		
CPT	48	11.1
INH	164	38.0
Both CPT and INH	69	16.0
No prophylaxis	151	35.0
Adherence to ART		
Good	317	73.4
Fair	69	16.0
Poor	46	10.7
Referral site		
Within facility	315	72.9
Outside the facility	117	27.1
Baseline functional status		
Working	366	84.7
Ambulatory	58	13.4
Bedridden	8	2.0
Baseline WHO clinical stage		
I	310	71.7
II	58	13.4
III	40	9.3
IV	24	5.6
Baseline BMI		
Under weight	112	25.93
Normal	268	62.04
Overweight or obese	52	12.04
Past opportunistic infection		
Yes	110	25.5
No	322	74.5

*ASM* Appointment spacing model, *TB* tuberculosis, *HIV* human immunodeficiency virus, *WHO* World Health Organization, *BMI* Body Mass Index, *ART* antiretroviral therapy

### A predictive model for LTFU among clients on ART

For the prediction of LTFU, the patient’s socio-demographic, personal and behavioral, clinical, and system-related prognostic determinants were considered. In the bi-variable binary logistic model, among the considered determinants: HIV disclosure status, prophylaxis status, ASM status, functional status, date of initiation for ART, educational status, adherence status, age, partner HIV status, caregiver status, residence, BMI, WHO clinical staging, HVL status, and marital status were significant predictors of LTFU and were fitted to the multivariable binary logistic regression model. While in the multivariable binary logistic regression model: not on prophylaxis status, on ASM model, being HVL, poor drug adherence, rural residence, and normal BMI were the remaining significant predictors of LTFU (Table 3).

**Table 3:** Prognostic determinants of LTFU among clients on ART in Gondar Town from October 2016 to April 2021

Prognostic variables	LTFU		Bi-variable	Multivariable
	No	Yes	COR (95% CI)	AOR (95% CI)
Partner HIV status				
Known status	111	16	1	
Unknown status	210	95	3.14 (1.81–5.77)	
Prophylaxis status				
Yes	244	37	1	1
No	77	74	6.34 (3.99–10.23)	2.82 (1.58–5.03)**
ASM				
Not ASM	71	4	1	1
ASM	250	107	7.59 (3.05–25.40)	4.92 (1.52–15.94)*
Adherence status				
Good	270	47	1	1
Poor	51	64	7.21 (4.48–11.74)	3.07 (1.70–5.57)***
Date of ART initiation				
Same day	189	80	1	
Not same day	132	31	0.55 (0.35–0.89)	
Marital status				
Married	146	25	1	
Unmarried	175	86	2.87 (1.75–4.72)	
HIV disclosure status				
Yes	156	80	1	
No	165	31	0.37 (0.23–0.59)	
BMI				
Underweight	89	23	1	1
Normal	191	77	1.56 (0.92–2.65)	2.75 (1.43–5.30)
Obese	41	11	1.04 (0.46–2.33)	
Residence				
Urban	265	86	1	1
Rural	56	25	1.44 (0.83–2.42)	2.20 (1.11–4.39)*
Care giver				
Yes	298	91	1	
No	23	20	2.84 (1.48–5.42)	
HVL status				
Not HVL	253	29	1	1
HVL	68	82	10.52 (6.45–17.59)	4.54 (2.43–8.47)***

^***^ = Significant at p-value < 0.001, * = significant at p-value < 0.05

*COR* Crude odds ratio, AOR adjusted odds ratio, *CI* confidence interval, *LTFU* lost to follow-up, *ASM* Appointment spacing model, *TB* tuberculosis, *HIV* human immunodeficiency virus, *WHO* World Health Organization, *BMI* Body Mass Index, *ART* antiretroviral therapy, *OI* opportunistic infection, *HVL* high viral load

After the multivariable logistic model, a total of six prognostic determinants were left for the prediction of LTFU and the relative contribution of each prognostic determinant to the probability of LTFU was calculated by dividing each beta coefficient by the lowest beta coefficient and rounding to the nearest integer (score chart rule formula) (Table 4).

**Table 4:** Reduced model prognostic determinants

Prognostic determinants	AOR (95% CI)	Regression coefficient	Contribution to risk score
Not on prophylaxis	2.82 (1.58–5.03) ***	1.03	1
On ASM model	4.92 (1.52–15.94) **	1.59	2
Normal BMI	2.75 (1.43–5.30) **	1.01	1
Poor adherence status	3.07 (1.70–5.57) ***	1.12	1
Being HVL	4.54 (2.43–8.47) ***	1.51	2
Residence (out of Gondar Town)	2.20 (1.11–4.39) *	0.79	1
Constant	− 4.70		

Significant codes: 0 ‘***’ 0.001 ‘**’ 0.01 ‘*’

*ASM* Appointment Spacing Model, *BMI* Body Mass Index, *HVL* high viral load

Therefore, the probability of LTFU among clients on ART using the regression formula was: Linear predictor of the model (lp) = − 4.70 + 1.03 * not on prophylaxis + 1.59 * on ASM + 1.12 * Poor adherence status + 1.51 * being HVL + 0.79 * residence (out of Gondar) + 1.01 * normal BMI.

Consequently, the probability of LTFU for each patient was predicted by the regression formula as follows, P (LTFU) = ${exp}^{(lp)} /(1+{exp}^{\left(lp\right)})$

Based on the regression formula, the probability of LTFU for each client on ART was calculated. Thus, using the Youden index value, the cut point for high and low risk for LTFU was determined to be 30.4%. Accordingly, 150 patients were at high risk for LTFU, and among those, 80 (53.3%) clients were lost from care. While 282 (65.3%) clients were at low risk of LTFU, about 31 (11%) were lost. The sensitivity and specificity of the probability of LTFU with the cut point of (30.4%), were 72.07% and 83.5%, respectively. The overall true prediction accuracy of the risk algorithm to predict LTFU was 76.62%, and the false prediction probability was 16.51% (Table 5).

**Table 5:** The prediction of LTFU using the reduced regression formula among HIV clients on ART in Gondar, 2021

Risk category	Percentage	Prediction of LTFU					
		Number of patients on ART	Incidence of LTFU	SN (%)	SP (%)	PPV (%)	NPV (%)
Low risk	< 30.4	282 (65.28%)	31 (11.0%)	72.07	83.49	60.15	89.63
High risk	≥ 30.4	150 (34.72%)	80 (53.33%)				
Total	89.20	432 (100%)	111 (25.69%)				

*SN* Sensitivity, *SP* Specificity, *PPV* positive predictive value, *NPV* negative predictive value

**Fig. 1:**
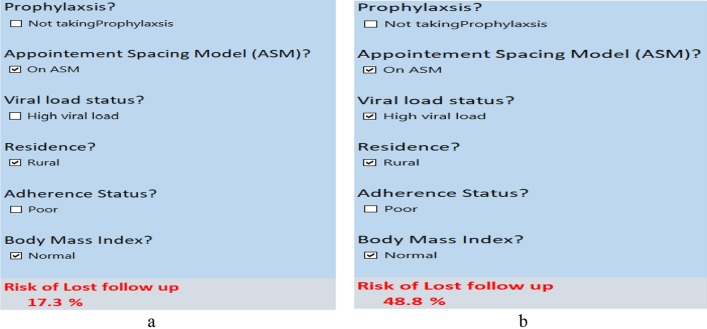
Risk prediction calculator for LTFU among HIV patients on ART in Gondar, as shown in an excel spreadsheet

A risk prediction tool has been generated as an excel spreadsheet that can be used in the clinic set up to perform these calculations automatically using information entered about patient’s risk determinants (Additional file 1). To illustrate, consider a patient from a rural setting, who has normal BMI, and is on ASM. Putting this value into the excel spreadsheet gives an estimated probability of LTFU of 17.3% (as shown in Fig. 1a) which is classified as low risk for LTFU. However, if another patient with the same characteristics as the previous patient but with another risk factor of being HVL, adding this information in the tool, gives a revised probability of 48.8% (as shown in Fig. 1b) which turns the patient risk classification from low risk into high risk of LTFU.

### Discrimination and calibration ability of the reduced model

**Fig. 2:**
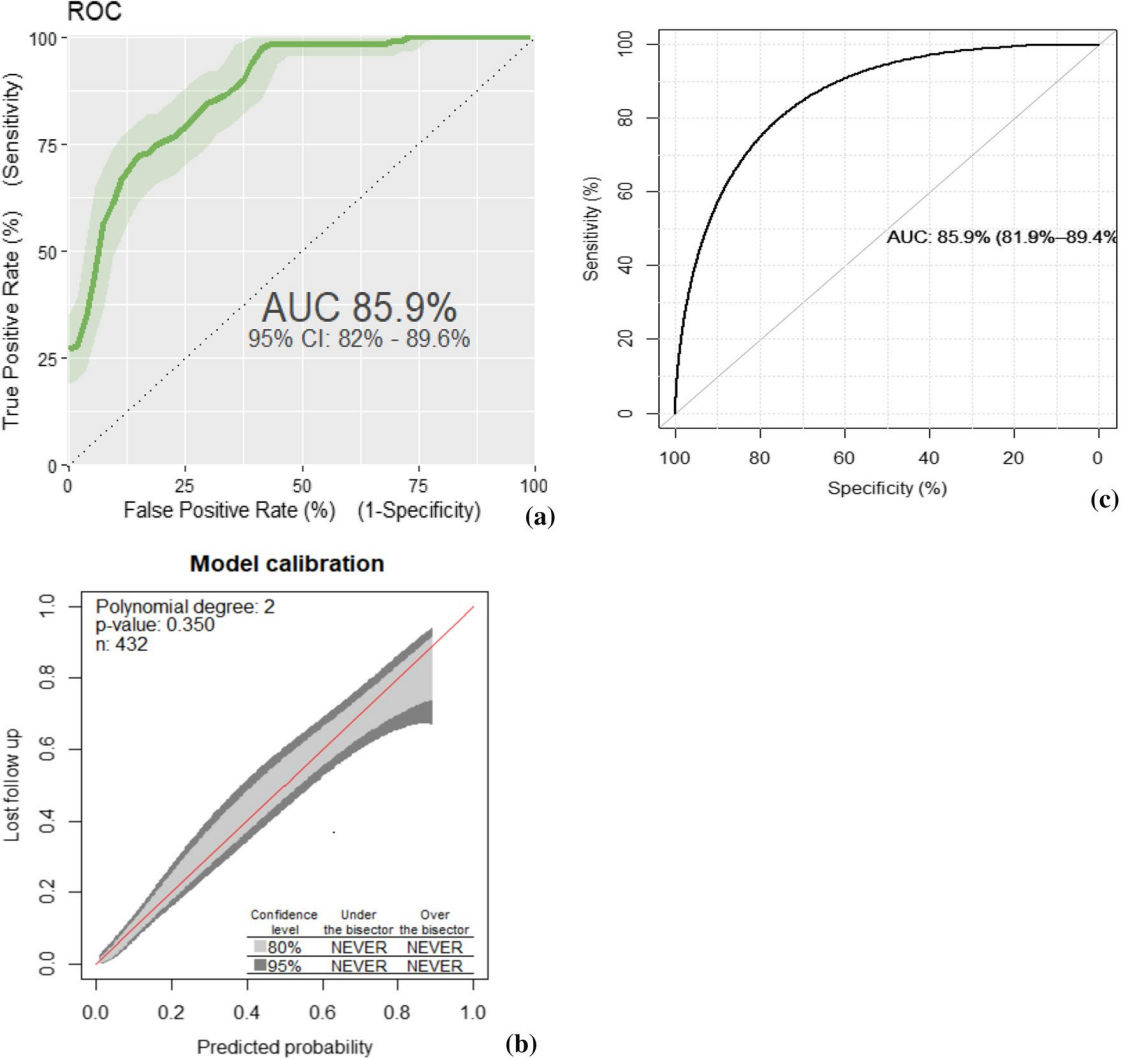
**a** AUROC for the reduced model, **b** model calibration plot, **c** Internal validation of the model using bootstrap for the LTFU prediction model

The final reduced model discriminative probability was assessed using the AUROC which was 85.9% (95% CI 82.0–89.6) (Fig. 2a). The internal validation of the model was checked through bootstrapping technique by drawing with replacement from the original sample. After 10,000 stratified bootstrap replicates, the AUC was 85.9% with the 95% CI (81.9–89.4%) (Fig. 2c). The model calibration was checked by comparing the agreement between the predicted probability of LTFU against the observed frequency using a calibration plot (p-value = 0.350) (Fig. 2b).

### Decision curve analysis of the model

**Fig. 3:**
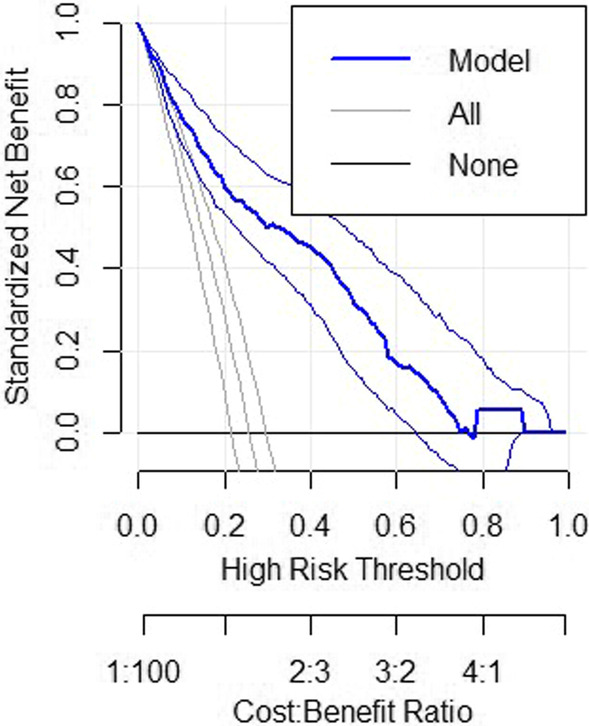
Decision curve analysis for prediction of LTFU among HIV clients on ART

Using the model for the prediction of LTFU has a better cost–benefit ratio as shown in Fig. 3. The prognostic model gives highest net benefit. If we take 0.2 risk threshold the net benefit for follow all will be around 0.2. This implies with less risk threshold have less net benefit however that incurs more cost. With the same risk threshold following based on the prognostic model the net benefit will around 0.6, which is low cost.

### Clinical prediction and decision rules for LTFU among HIV clients on ART

**Fig. 4:**
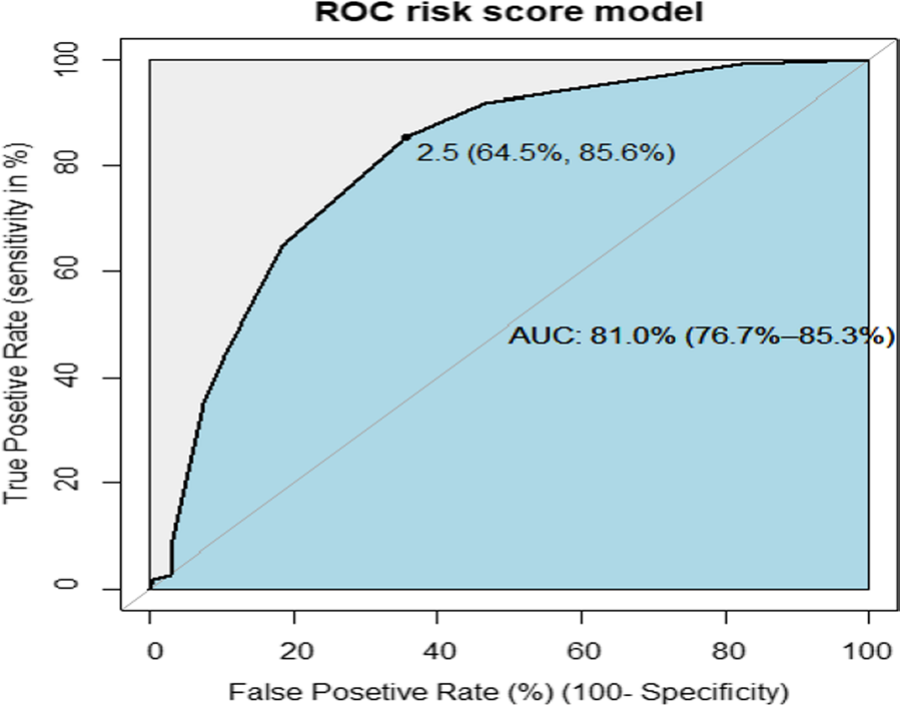
AUROC for risk of LTFU for HIV clients on ART using risk score chart rule

For the ease of clinical applicability, a score chart rule was developed and with that, the prediction of the risk score tool had eight scores with the AUROC of the simplified risk score 81.0% (95% CI 76.7–85.3%) (Fig. 4). The sensitivity, specificity, positive predictive value, and negative predictive value of each risk score category was also determined (Additional file 2).

For better clinical decision rule, the risk score was categorized as low and high risk of LTFU. The risk score cut point was declared using Youden’s index value, which is the maximum sensitivity and specificity of the risk score. At this risk score value (2.5), the sensitivity and specificity of the risk score AUROC curve were maximized, which is, 64.5% and 85.6%, respectively. Therefore, the individual prediction of LTFU was a high risk if the patient has a risk score value of more than and equal to three (after rounding to the nearest integer).

Based on the risk score category developed, 86 patients had a risk score of less than 3 (low risk). Three hundred forty-six patients had a risk score of more than or equal to 3 (high risk), among them, 110 (31.8%) patients sustained LTFU from care. The sensitivity and specificity of the risk algorithm category were 64.5% and 85.6%, respectively. The positive and negative predictive values of the risk category were 45.45% and 92.82%, respectively. The overall true prediction accuracy of the risk algorithm to predict LTFU among clients on ART was 82.41%, and the false-positive rate was 17% (Table 6).

**Table 6:** Prognostic risk classification of LTFU among HIV clients on ART in Gondar town using simplified prediction risk score among 432 clients

Risk category	Score range	Prediction of LTFU					
		Number of HIV patients on ART	Incidence of LTFU	SN (%)	SP (%)	PPV (%)	NPV (%)
Low risk	< 3	86 (19.91%)	1 (1.16%)	85.59	64.49	45.45	92.82
High risk	≥ 3	346 (80.09%)	110(31.39%)				
Total	8	432 (100%)	111 (25.69%)				

*SN* Sensitivity, *SP* Specificity, *PPV* positive predictive value, *NPV* negative predictive value

Finally, each patient’s risk for LTFU on HAART was predicted using the score chart formula.

Probability of LTFU = (1 * not on prophylaxis) + (2 * on ASM) + (1 * poor adherence) + (2 * HVL) + (1 * residence, out of Gondar) + (1 * Normal BMI).

## Discussion

This is the first prognostic research on LTFU among HIV clients on ART. This study revealed the incidence of LTFU was 11.19 (95% CI 8.95–13.99) per 100-person years. One in 4 patients (25.69%) had LTFU from the treatment in the current study. When compared to findings elsewhere in Ethiopia, the LTFU rate in the present study was higher than findings from Aksum [9], Debre Markos [17], Mizan Teferi [24]. It was similar to results from Gondar [21, 25], South Ethiopia [22], Hadiya [27] but lower than the findings from Eastern Ethiopia (Jigiga) [11]. In addition, this rate was lower than findings from studies conducted in South Africa [36], Malawi [37], and Guinea-Bissau [38]. The dissimilarity in measurement [39], access to HIV care services, innovation, adoption of new strategies like the universal test and treat approach [40], and difference in year of study could be the possible reasons for variations in rates of LTFU.

In previous years, the focus of the research was to explain the incidence and factors associated with a certain outcome. But in recent years, the emphasis is shifted to predicting the risk using a combined set of characteristics. In our study, a combination of six prognostic determinants (prophylaxis status, ASM status, HVL status, adherence level, residence, BMI status) results in an AUROC of 0.86 (95% CI 0.8–0.9), which is good accuracy according to diagnostic accuracy classification [41, 42]. Having an AUROC of 0.86 (95% CI 0.8–0.9) means that the model is 86% accurate in discriminating between a randomly selected subject who was lost from a randomly selected subject who was not lost from care.

Among the prognostic determinants, HVL status alone has the highest AUC value which is 0.76% (95% CI 0.72–0.81%) followed by prophylaxis and adherence status with an AUC value of 0.71% (95% CI 0.66–0.76%) and 0.71% (95% CI 0.66–0.76%), respectively. Other’s determinants have low predictive value which is less than 0.70% [ASM status 0.59% (95% CI 0.56–0.62%), normal BMI 0.55% (95% CI 0.56–0.62%), and residence 0.53% (95% CI 0.50–0.60%)].

Though both the regression formula and risk score chart have good accuracy, the AUROC from the regression formula is slightly higher than that of the risk score 0.86 (95% CI 0.8-0.9) vs 0.81 (95% CI 0.77–0.85). Thus, using the regression formula to predict LTFU is better and advisable. The model has also a good calibration with a p-value of 0.350. Good calibration means that the estimated probability of LTFU using the model is similar to the observed LTFU frequency. A statistically significant (p < 0.05) test indicates marked differences between predicted probabilities and observed once and thus poor calibration.

As shown in Fig. 3, the model has the highest net benefit across the entire range of threshold probabilities, which indicates that the model has the highest clinical and public health value. Hence, using the model for the prediction of LTFU has a higher net benefit than not using it. Prognostic research aims to find a risk prediction tool that is simple to use, accurate in predicting risk, generalizable across contexts, and uses routinely collected determinants that are needed to identify patients at high risk for poor outcomes and to provide individualized risk assessment [32]. Thus, clinicians can also use the developed risk score chart for the prediction of LTFU among ART patients as it is simple and has good prediction accuracy (AUC = 81%).

As a result, the overall risk score for the risk prediction tool based on the score chart is 8, and the risk of LTFU grows as the risk score increases. We categorized the cohort into two risk groups in addition to predicting the degree of LTFU risk associated with each risk score. When compared to the low-risk group (risk score less than 3), those in the high-risk category (risk score greater than or equal to 3) had a fourfold (OR 3.68; % CI 1.69–5.66) increased risk of LTFU.

Depending on the availability of resources, health care providers can use different cutoff points. If the providers value sensitivity and specificity equally, the risk score’s cutoff value of 3 maximized the value of both sensitivity and specificity (86% and 64%, respectively). The positive and negative predictive values, respectively, were 45% and 93%. However, health care providers may choose to utilize different cutoff points depending on the importance of false positives and false negatives. A lower risk score cutoff value would target a substantial section of our population for intervention and identify the majority of people who were lost to therapy.

Patients who do not take prophylaxis were found to be at higher risk of LTFU. This was consistent with other studies [21, 24, 25, 27, 28, 31]. This is because of the direct effect of isoniazid in preventing active tuberculosis, which in turn improves the quality of life of patients, which leads to a longer stay in the treatment [21, 25, 27]. The exiting intervention such as management and prevention of opportunistic infections like pneumocystis pneumonia (PCP), toxoplasmosis, bacterial infections and diarrheal diseases through providing prophylaxis like CPT could encourage patients to be engaged and could bring the effort to retain patients from the start of HIV treatment [6, 21, 43].

Patients with suboptimal adherence were at an increased risk of being LTFU when compared with those with exemplary commitment. This was supported by other studies [8, 25, 27]. The possible reason could be patients with suboptimal adherence may have socio-demographic and clinical problems that affect their adherence initially, which further affect retention in care [44]. In addition, patients with suboptimal adherence are at a higher risk of treatment failure, which makes them to be more vulnerable to many opportunistic diseases, with higher chance to have more pill burden, adverse drug toxicities, and interactions among opportunistic infection treatment and ART, which demands a high level of commitment to follow all those medications [44, 45].

This study revealed that rural residents were found to be more likely to be LTFU in the treatment as compared to their counterparts. Studies evidenced that travel time to the clinics and its opportunity costs (in terms of financial cost or time allocated to something else), level of patient’s awareness of the treatment, and social stigma are significant barriers to patient adherence to ART and maintenance in care [18, 37].

Contrary to previous evidences [37, 38, 46], patients who had a normal baseline BMI were about three times more likely to be LTFU in treatment compared to those patients who had low BMI. This may be due to the reason that patients who had a normal baseline BMI may feel that they are well and their health-seeking behavior may be inadequate and patients with low BMI at ART initiation were probably more symptomatic and had counseled about good adherence in the lifelong follow-up treatments, which may have resulted in greater motivation to remain in care [47].

In our study, patients on ASM are at a higher risk of LTFU than patients not on ASM. This may be due to the problem with lower potency of the drug, which may happen due to poor handling of several ART medications that patients have to take for 6 months as well patients may not disclose their HIV status and thus worried about keeping too many numbers of pills at home without being seen which may have an impact on their adherence [48]. The other reason may be, that patients may not be screened well using the criteria for ASM which patients may have an unseen deadly opportunistic infection like Tuberculosis, a cryptococcal infection that can take the life of such patients. Due to these reasons, patients may be lost from care.

LTFU was also more prevalent in individuals who had HVL in the follow-up. Patients with HVL were almost five times at increased risk of being lost. This is because patients with HVL in the follow-up period are more likely to have problems with adherence, psychosocial issues like fear of stigma, lack of social support, mental illness, substance abuse, poor livening condition, or even primary drug resistance, which have a direct or indirect effect on the continuity of care [43, 49].

This study has some limitations and strengths. Our research is innovative in that it uses routinely collected patient data available in many HIV programs in resource-limited settings, which allows for the model to be used across diverse backgrounds. The model is pragmatic in that it is a simple point-based model that can be calculated by various health care professional’s ranging from providers to adherence counselors. It can be easily adapted to mobile-app technology or the existing electronic medical record (smart care) so that HIV program could use this risk score to identify patients at the highest risk of LTFU after starting treatment and provide such patients with differentiated models of HIV care and interventions to reduce LTFU and the grave consequences following it.

Despite the strengths of this simple risk prediction score model, several limitations need to be acknowledged. First, though the model has good discrimination and calibration in the bootstrapped samples, the model should undergo external validation to see the performance of the risk prediction model/score in other populations. Second, is that we did not include determinants like monthly income, cigarette smoking, alcohol, and substance abuse, hemoglobin level, pain status, Hepatitis B and C status, baseline CD4 count, drug regimen, and adverse drug reactions which could have an impact on LTFU and maybe essential determinants for prediction of LTFU. Thus, the model prediction cannot be extended to such patients, limiting the model’s applicability. Third, the impact of Covid-19 on LTFU among HIV patients on ART was not assessed due to the retrospective nature of the study. Last, our study included people with a new and existing HIV diagnosis. People with a new HIV diagnosis may have different challenges like disclosure, fear of stigma, adherence issues, and modification of life to stay in care. Therefore, they may need a different risk score.

## Conclusion and recommendation

The incidence of LTFU among clients on ART in Gondar town was high (> 3%). we have identified a set of readily available tools that can be used to predict LTFU among HIV patients on ART. The predictors of LTFU were being HVL, on ASM, not being on prophylaxis, poor drug adherence, rural residence, and normal BMI. The tool has good accuracy and discriminative ability. In addition, the tool can be used to stratify PLHIV into risk groups that can be identified for targeted intervention. In settings with similar demographics, the risk prediction tool can assist clinicians and health care providers to identify high-risk individuals for LTFU and target interventions. Researchers should externally validate the developed prediction model for the applicability of the clinical setup.

## Supplementary Information

**Additional file 1.** Risk prediction calculator for calculation of probability of LTFU among HIV patients on ART in Gondar, Ethiopia.

**Additional file 2.** Performance of the risk scores at different cutoff points.

## Data Availability

All data analyzed during this study are included in this published article [and its additional information files].
