# Supplementary material for: Development and validation of a risk prediction model for lost to follow-up among adults on active antiretroviral therapy in Ethiopia: a retrospective follow-up study
Source: BMC Infect Dis. 2022 Sep 7;22:727. doi: 10.1186/s12879-022-07691-x (PMC9449961; doi:10.1186/s12879-022-07691-x)
Supplement: Supplementary file 2 — Additional file 2. Performance of the risk scores at different cutoff points. [file 12879_2022_7691_MOESM2_ESM.docx]

Additional file 2: Table S1. Performance of the risk scores at different cutoff points.

| Cutoff point | Sensitivity (%) | | Specificity (%) | PPV (%) | NPV (%) | |  |
| --- | --- | --- | --- | --- | --- | --- | --- |
| 0 | 100 | 0.00 | | 25.69 | | 98.28 | |
| 1 | 99.10 | 17.76 | | 29.41 | | 95.00 | |
| 2 | 91.90 | 53.27 | | 40.48 | | 92.83 | |
| 3 | 85.59 | 64.49 | | 45.45 | | 87.04 | |
| 4 | 64.86 | 81.62 | | 54.96 | | 82.29 | |
| 5 | 44.14 | 89.72 | | 59.76 | | 80.49 | |
| 6 | 35.13 | 92.52 | | 61.90 | | 75.49 | |
| 7 | 9.01 | 96.88 | | 50.00 | | 74.29 | |
| 8 | 2.70 | 97.20 | | 25.00 | | 74.53 | |

* Sum of the risk score. PPV: Positive Predictive Value; NPV: Negative Predictive Value

**Additional file 2.**

- **Patient classification based on their care needs in the Ethiopian context:**
  - **Category 1a:** People, who present when, well, potentially with higher CD4 cell counts.
  - **Category 1b:** Pregnant/Breast feeding women with no other complications, rapidly growing children (0–5 years old) and adolescents with no advanced disease or other complications.
  - **Category 2:** People with advanced disease are defined as those presenting to care with a CD4 count below 200cells/mm3 or WHO disease stages 3 and 4.
  - **Category 3:** Those who are already on ART but need careful monitoring to ensure timely action as required (For example treatment failure suspects, patients with other chronic comorbidities, patients with identified adherence barriers).
  - **Category 4:** Stable individuals are defined as those who have received ART for at least one year and have no adverse drug reactions that require regular monitoring and have good understanding of lifelong adherence and evidence of treatment success.
    - Evidence of treatment success can be seen using:
      - Two consecutive viral load measurements below 1000 copies/mL with no current illnesses, excluding children, adolescents, pregnant and lactating women. OR
      - In the absence of viral load monitoring, rising CD4 cell counts or CD4 counts above 200cells/mm3, an objective adherence measure either from the client’s self-report or by doing pill count, can be used to indicate treatment success.
